# Comparative Transcriptome Profiling of *Gaeumannomyces graminis* var. *tritici* in Wheat Roots in the Absence and Presence of Biocontrol *Bacillus velezensis* CC09

**DOI:** 10.3389/fmicb.2019.01474

**Published:** 2019-07-09

**Authors:** Xingxing Kang, Yu Guo, Shuang Leng, Lei Xiao, Lanhua Wang, Yarong Xue, Changhong Liu

**Affiliations:** ^1^State Key Laboratory of Pharmaceutical Biotechnology, School of Life Sciences, Nanjing University, Nanjing, China; ^2^School of Chemical Engineering and Technology, China University of Mining and Technology, Xuzhou, China

**Keywords:** endophytic bacteria, pathogenic fungi, phytopathology, RNA sequencing, wheat disease

## Abstract

This study aimed to explore potential biocontrol mechanisms involved in the interference of antagonistic bacteria with fungal pathogenicity *in planta*. To do this, we conducted a comparative transcriptomic analysis of the “take-all” pathogenic fungus *Gaeumannomyces graminis* var. *tritici* (*Ggt*) by examining *Ggt*-infected wheat roots in the presence or absence of the biocontrol agent *Bacillus velezensis* CC09 (*Bv*) compared with *Ggt* grown on potato dextrose agar (PDA) plates. A total of 4,134 differentially expressed genes (DEGs) were identified in *Ggt*-infected wheat roots, while 2,011 DEGs were detected in *Bv*+*Ggt*-infected roots, relative to the *Ggt* grown on PDA plates. Moreover, 31 DEGs were identified between wheat roots, respectively infected with *Ggt* and *Bv*+*Ggt*, consisting of 29 downregulated genes coding for potential *Ggt* pathogenicity factors – e.g., para-nitrobenzyl esterase, cutinase 1 and catalase-3, and two upregulated genes coding for tyrosinase and a hypothetical protein in the *Bv*+*Ggt*-infected roots when compared with the *Ggt*-infected roots. In particular, the expression of one gene, encoding the ABA3 involved in the production of *Ggt*’s hormone abscisic acid, was 4.11-fold lower in *Ggt*-infected roots with *Bv* than without *Bv*. This is the first experimental study to analyze the activity of *Ggt* transcriptomes in wheat roots exposed or not to a biocontrol bacterium. Our results therefore suggest the presence of *Bv* directly and/or indirectly impairs the pathogenicity of *Ggt* in wheat roots through complex regulatory mechanisms, such as hyphopodia formation, cell wall hydrolase, and expression of a papain inhibitor, among others, all which merit further investigation.

## Introduction

“Take-all” is one of the most severe soil-borne diseases of wheat plants worldwide, caused by the necrotrophic fungus *Gaeumannomyces graminis* var. *tritici* (Bithell et al., 2016). This pathogen infects healthy wheat roots via infectious hyphae that penetrate the cortical cells of the root and progress upward into the stem base. Because this process invariably disrupts water flow, it eventually results in the premature death of infected plants. However, since the well-known virulence mechanism of *Ggt* might contribute to improved control of this fungal pathogen, considerable research effort has sought to better understand the mechanisms underlying *Ggt* pathogenicity (Dori et al., 1995; Yu et al., 2010; Yang et al., 2012). Consequently, many genes that contribute to *Ggt* pathogenicity have been identified, such as cellulase, endo-β-1,4-xylanase, pectinase, xylanase, β-1,3-exoglucanase, glucosidase, aspartic protease, and β-1,3-glucanase of cell wall degrading enzymes (CWDEs) (Yang et al., 2015). Based on their comparative transcriptome analysis of *Ggt* in axenic culture and *Ggt*-infected wheat, Yang et al. (2015) recently pointed out that many genes related to signaling, penetration, fungal nutrition, and host colonization are highly expressed during *Ggt* pathogenesis in wheat roots. Nevertheless, because of the complexity of the interaction between *Ggt* and its host plants, the pathogenesis of *Ggt* in wheat roots remains unclear. Additionally, control of the disease is hindered by a lack of resistant varieties and environmentally friendly fungicides.

Several studies have shown that beneficial bacteria, such as *Bacillus subtilis* (Liu et al., 2009, 2011; Durán et al., 2014; Yang et al., 2018), *Bacillus velezensis* (Wu et al., 2012; Luo et al., 2013; Kang et al., 2018), and *Pseudomonas fluorescens* (Daval et al., 2011; Kwak and Weller, 2013; Lagzian et al., 2013; Yang et al., 2014, 2017), could be used as effective and eco-friendly biocontrol agents to protect wheat from take-all disease. Among them, *B. velezensis* is a newly reported species that may be used to control take-all and other fungal diseases, such as spot blotch and powdery mildew (Cai et al., 2017; Kang et al., 2018). More specifically, *B. velezensis* CC09 (*Bv*) is an endophytic biocontrol bacterium, originally isolated from healthy *Cinnamomum camphora* leaves, that has broad antifungal spectra against many phytopathogens (Cai et al., 2016). It possesses several key biocontrol traits, namely, the production of strong antifungal metabolites (e.g., iturins and fengycins), promotion of plant growth, and induction of plant resistance (Cai et al., 2017; Kang et al., 2018). Recently, we found that this strain can cause the swelling, deformation, and cell content release of *Ggt* mycelia *in vitro*, inhibiting *Ggt* mycelia density and spread in wheat (Kang et al., 2018). Moreover, we also found that *Bv* could colonize and migrate in plants, leading to a 66.67% disease-control efficacy of take-all and 21.64% of spot blotch, with a single treatment inoculated on roots (Kang et al., 2018). These attributes make *Bv* a promising biocontrol agent for the long-term and effective protection of wheat from soil-borne and leaf diseases.

Yet, despite these established biocontrol features of *Bv*, limited information is available concerning its direct and indirect effects upon *Ggt*’s fungal pathogenicity *in planta*. For example, how *Ggt* responds to the presence of *Bv* in *Ggt*-infected plants remains unknown. In this study, we performed an RNA sequencing (RNA-Seq) analysis of *Ggt* in wheat roots with and without *Bv*. Since the *Ggt* transcriptome in axenic culture and *Ggt*-infected wheat roots differed markedly and varied during the infection process (Yang et al., 2015), the transcriptome of *Ggt* grown on potato dextrose agar (PDA) plates was also determined to serve as the control (*CK*). Through a comparative analysis of *Ggt* transcriptomes under three different conditions (e.g., grown on PDA, within wheat roots in the presence or absence of *Bv*), we sought to reveal the possible pathogenicity gene(s) of *Ggt* and its regulation by *Bv in planta* during the early infection of wheat roots.

## Materials and Methods

### The Wheat, Bacterium, and Fungus

The winter wheat (*Triticum aestivum* “Sumai 188”) used in this study was purchased from the Jiangsu Academy of Agricultural Sciences, Nanjing, China. The endophytic bacterium *Bv* was isolated from *C. camphora* leaf tissue (Cai et al., 2016) and deposited in the China Center of Industrial Culture Collection (No. CICC24093). The genome sequence of *Bv* was deposited in the GenBank database under accession number CP015443. *Bv* was cultured in LB medium at 37°C and 200 rpm for 12 h (exponential growth phase), and then harvested by centrifugation at 8,000 rpm for 10 min at 4°C, and finally resuspended in distilled water to a final concentration of 1.0 × 10^8^ CFU/mL (Kang et al., 2018). The take-all causative pathogen, *Gaeumannomyces graminis* var. *tritici* strain Ggt-C2 (*Ggt*), was deposited in Agricultural Culture Collection of China (No. ACCC 30310); it was a gift from Prof. Jian Heng (Department of Plant Pathology, Chinese Agricultural University, Beijing, China). The pathogenicity of *Ggt* was evaluated on *T. aestivum* Sumai 188 in our prior study (Kang et al., 2018). The periphery of 10-day-old colonies of *Ggt* on PDA plates at 25°C was used for inoculations.

### Root Inoculation and Sampling

Seeds of winter wheat were surface disinfected and germinated, as described by Kang et al. (2018). Germinated wheat seeds were cultured on a sterilized 72-cell seedling tray containing 120 mL of 1/2 MS (Duchefa Biochemie, Haarlem, Netherlands, catalog number M022250) and incubated at 25°C under a 14-h light/10-h dark cycle for 7 days. The roots of 15 7-day-old seedlings were inoculated with 30 mL of *Bv* inoculum (1.0 × 10^8^ CFU/mL). An equal amount of sterile distilled water was used to treat the roots, as a negative control. Five days after inoculation with *Bv*, the seedlings were gently removed from the tray, their roots were rinsed with sterile water, and then they were placed on water agar plates. Half of the seedling roots, whether inoculated with *Bv* or not, were fully covered by the fresh periphery of the 10-day-old colonies of *Ggt* and maintained for 3 days in a plant growth chamber at 25°C, under 50% relative humidity and a 14-h light/10-h dark cycle (light intensity of 200 μmol m^–2^ s^–1^). At this time, the mycelium had invaded the root cortex of wheat roots, but these lacked obvious symptoms (Kang et al., 2018). Approximately 15 seedling roots were pooled for each biological replicate of the *Ggt* or *Bv*+*Ggt* treatment; they were immediately flash frozen, and stored in liquid nitrogen until later usage, while 0.1 g of the periphery of the 10-day-old *Ggt* colony was remove from PDA plates and maintained in liquid nitrogen as well. Assays were repeated for three independent experiments. Thus, the total number of seedlings used was 45 (three replicates) per treatment or control group.

### RNA-Seq and Data Analysis

Total RNA was extracted from *Ggt*- and *Bv*+*Ggt*-infected roots of wheat seedlings using an RNAiso Plus kit (TaKaRa, Otsu, Japan). The same method was used to extract total RNA from *Ggt* (periphery of 10-day-old colony) grown on PDA plates. The purity and integrity of the total RNA were determined using an Agilent 2100 Bioanalyzer RNA chip (Agilent Technologies, Santa Clara, CA, United States). The mRNA was purified from 3 μg of total RNA per sample using oligo(dT) magnetic beads and then cleaved into short fragments using divalent cations under elevated temperature. The short fragments were used for first-strand cDNA synthesis by using random primers and reverse transcriptase (Invitrogen, Carlsbad, CA, United States), followed by a second-strand cDNA synthesis, performed using DNA polymerase I and RNaseH. After the end repair process and ligation of adaptors, these second-strand cDNA products were purified and amplified via polymerase chain reaction (PCR) to create the final cDNA library.

The cDNA libraries were sequenced on an Illumina HiSeq^TM^ 4000 platform by following the default Illumina Stranded RNA protocol (Personalbio, Shanghai, China). Clean reads were obtained by removing adapter sequences, any reads with more than 10% N, along with low-quality sequences (e.g., more than 50% of each read that had a Phred score Q ≤ 5). The Q20, Q30, and GC contents of the cleaned data were calculated (Yang et al., 2015). Each sample resulted in approximately 17 million 150-bp clean reads (sequencing data >2 gigabases per sample) for *Ggt* on PDA plates and 180 million 150-bp clean reads (sequencing data >25 gigabases per sample) for *Ggt*- or *Bv*+*Ggt*-infected root sample. Filtered clean reads were aligned to the reference *Ggt* genome in the genome website^1^ using SOAPaligner/SOAP2 (Li et al., 2009). All RNA-Seq data generated for this study were deposited in the National Center for Biotechnology Information Sequence Read Archive under BioProject IDs PRJNA485739 and PRJNA496308. Reads per kilobase per million (RPKM) were used to normalize the levels of gene expression for each replicate. To evaluate the reproducibility of RNA-Seq, a hierarchical cluster analysis (HCA) was done using the command “heatmap3::heatmap3” in the R (v3.5.3) package “gplots” (Warnes et al., 2015) with the hclust command (R Core Team, 2009). The “DESeq” package (1.10.1) of R was used to analyze differentially expressed genes (DEGs) in *Ggt* among the three conditions under the criteria of *P* values < 0.05 and an absolute log2 ratio ≥1 (Anders and Huber, 2012).

### Functional Analysis of RNA-Seq Data

Two enrichment analyses of DEGs between samples were performed, topGO, and KEGG (Kyoto Encyclopedia of Genes and Genomes), by respectively, using the AgriGO analytical tools^2^ and the KEGG website^3^ (Alexa and Rahnenfuhrer, 2010; Yang et al., 2015). GO terms and KEGG pathways were considered significantly enriched by DEGs if the *P* values were < 0.05. All Venn diagrams were produced using Venny Tools^4^. Pathogenesis-related genes were identified through a BLAST search of the pathogen–host interaction (PHI) database (identity >25, *E*-value: 1e-10) (Jing et al., 2017).

The STEM (short time-series expression miner) software^5^ (Ernst et al., 2005) was used to identify the significantly enriched expression profiles (*P* values < 0.05) in *Ggt* on PDA plates and in *Ggt*- and *Bv*+*Ggt*-infected wheat roots. The log2-transformed RPKM values of *Ggt* on plates and in *Ggt*-infected roots in the absence or presence of *Bv* were used as the input data set. The software parameters for this STEM analysis were as follows: maximum number of model profiles = 8; maximum unit change in model profiles between treatments = 2; and calculated method of significance level = permutation test corrected by Bonferroni correction.

### Validation of RNA-Seq Results via Quantitative Reverse Transcription PCR

The expression levels of six pathogenicity DEGs were determined by using quantitative reverse transcription PCR (qRT-PCR) to confirm the prior results of the RNA-Seq analysis. Total RNA from *Ggt*- and *Bv*+*Ggt*-infected wheat roots was each reverse transcribed into cDNA with the PrimeScript^TM^ 1st Strand cDNA Synthesis kit (Takara, Dalian, China, Code No. 6110A) according to the manufacturer’s protocol. The qRT-PCR was carried out by an Applied Biosystems 7500 Real-Time PCR System (Applied Biosystems, Foster City, CA, United States) using a SYBR^®^ Advantage^®^ qPCR premix (Toyobo, Osaka, Japan). Each qRT-PCR was performed with a 20-μL volume containing 2 μL of cDNA, 0.4 μL of each primer (10 μM), 10 μL of 2 × SYBR Green PCR Master Mix, and 7.2 μL of nuclease-free water. The amplification went as follows: 95°C for 30 s, 40 cycles at 95°C for 5 s, and 60°C for 5 s. The qRT-PCR primers used for the DEGs’ validation are listed in Supplementary Table S1. Three housekeeping genes – encoding actin, tubulin beta, and elongation factor2-1 – served as internal reference for qRT-PCR. Each reaction was performed in triplicate independent experiments for the reference and selected genes. Gene expression was evaluated by applying the 2^–ΔΔCt^ method (Livak and Schmittgen, 2001).

### Statistical Analyses

The qRT-PCR amplification data are expressed here as mean ± standard deviation (SD) of at least three independent biological experiments. PRISM software v7.0 (Graph-Pad Software, San Diego, CA, United States) was used to perform one-way analysis of variance (ANOVA) that compared the three conditions. Tukey’s multiple pairwise comparison test was applied to the mean relative expression levels of selected pathogenicity genes (first normalized by the three internal reference genes). A *P*-value of less than 0.05 was deemed statistically significant.

## Results

### General Analyses of RNA-Seq Data

After removing the low-quality reads and adaptors, a total of 67,293,916, 553,791,342, and 553,453,160 clean reads were generated from the mRNA of *Ggt* on PDA and *Ggt* in wheat roots with and without *Bv*, which accounted for 91.6%, 83.2%, and 84.9% of raw reads, respectively (Supplementary Table S2). The quality of each library was similar, ranging from 97.02% to 98.63% of the raw reads with quality values of Q ≥ 20, and likewise from 92.16% to 96.03% of the raw reads with quality values of Q ≥ 30. Their average GC contents were 59.52%, 56.67%, and 57.77% for *Ggt* on PDA and *Ggt* in wheat roots with and without *Bv*, respectively. Together, these results confirmed the high quality of our sequencing data and their robust suitability for further analysis.

### Identification of DEGs

A total of 9,588, 9,389, and 8,826 expressed genes were, respectively detected in *Ggt* on PDA and in wheat roots in the absence and presence of *Bv*, which corresponded to 64.19%, 62.86%, and 59.09% of all genes (14,936) in the *Ggt* genome. We found 8,395 genes expressed in *Ggt* (RPKM > 1) under all three conditions. Based on the respective RPKM values of these 8,395 genes, HCA showed that the three biological replicates from each treatment clustered into an independent branch (Supplementary Figure S1), thus indicating RNA-Seq data were reliable, being highly repeatable between biological replicates. When compared with *Ggt* grown on PDA, 4,134 DEGs (2,142 upregulated, 1,992 downregulated) and 2,011 DEGs (957 upregulated, 1,054 downregulated) were identified in *Ggt* in wheat roots in the absence and presence of *Bv*, respectively (Supplementary Figure S2A). The total numbers of upregulated and downregulated genes in the *Ggt*-treated samples were, respectively, 2.24- and 1.89-fold higher than those observed in the *Bv*+*Ggt*-treated group. As the Venn diagram shows, 1,251 and 66 upregulated DEGs, as well as 1,000 and 62 downregulated DEGs, were uniquely found in *Ggt* in wheat roots without and with *Bv*, respectively (Supplementary Figure S2B). However, overall rates of gene expression were similar between these two treatments (Supplementary Figure S2C).

In addition, a total of 31 DEGs were detected between the *Ggt*- and *Bv+Ggt-*infected wheat root libraries, with two upregulated genes (GGTG_06400 and GGTG_05929) and 29 downregulated genes in *Bv+Ggt-*infected roots relative to *Ggt*-infected roots (Table 1). Among the downregulated DEGs in *Bv*+*Ggt*-infected wheat roots, 13 genes (41.94%) were associated with secreted proteins, three genes (9.68%) encoded pathogenicity proteins (para-nitrobenzyl esterase, cutinase 1, and catalase-3), two genes were linked to cell wall lysis enzymes (esterase and cutinase), and one gene encoded peroxidases (catalase-3). In particular, the expression of ABA3 protein-encoding gene involved in the biosynthesis of abscisic acid was downregulated 4.11 times in *Bv*+*Ggt*-infected wheat roots compared with *Ggt*-infected wheat roots; this suggested that *Bv* might inhibit mycelial infection by regulating *Ggt*-derived abscisic acid. All these results indicated that the presence of *Bv* in wheat root directly or indirectly reduced the expression of pathogenicity genes.

**TABLE 1:** The expression profile of *Ggt* DEGs that were only affected by *Bv in planta* based on the analysis comparing *Bv+Ggt* and *Ggt* transcriptomic data.

**Gene ID**	***Ggt***	***Bv+Ggt***	***Bv+Ggt_vs_Ggt***	**Protein name**	**Secreted protein**	**PHI**
GGTG_03282	5.29	2.90	−2.33	Para-nitrobenzyl esterase	Yes	PHI:2032
GGTG_10566	11.38		−4.80	Cutinase 1	Yes	PHI:2383
GGTG_10011			−2.38	Catalase-3	Yes	PHI:1034
GGTG_08754	4.39	1.79	−2.54	Endothiapepsin	Yes	
GGTG_06400		2.97	1.96	Tyrosinase	Yes	
GGTG_12447	3.94		−2.56	Arylsulfatase-like protein	Yes	
GGTG_06631	9.83		−3.93	Cell wall protein	Yes	
GGTG_05929	−2.09		1.42	Hypothetical protein	Yes	
GGTG_13651	10.54	8.25	−2.23	Hypothetical protein	Yes	
GGTG_07328	8.98	6.96	−1.96	Hypothetical protein	Yes	
GGTG_00233	12.22	9.88	−2.28	Diaminopimelate decarboxylase	Yes	
GGTG_08345	10.69	8.20	−2.44	Hypothetical protein	Yes	
GGTG_08028	10.15		−4.05	Cell wall protein	Yes	
GGTG_07717	5.68	3.97	−1.65	Copper-transporting ATPase 1		
GGTG_08722	4.03		−2.50	Sodium/phosphate symporter		
GGTG_07931	3.18	1.65	−1.47	Putative nucleosome assembly protein		
GGTG_12291	2.03		−1.53	2-hydroxyacid dehydrogenase		
GGTG_05152	3.04		−2.48	Arsenical-resistance protein		
GGTG_08620	3.95	2.36	−1.52	L-ornithine 5-monooxygenase		
GGTG_05151	3.94		−2.20	NADPH-dependent FMN reductase ArsH		
GGTG_02030	3.32		−2.13	Aldehyde reductase ii		
GGTG_02188		−1.62	−1.94	Glycosyl Hydrolase-glycosidase superfamily		
GGTG_03806	4.28		−2.32	NAD(P)-binding protein		
GGTG_09078	4.86		−4.59	ubiE/COQ5 methyltransferase		
GGTG_10994	4.11		−2.25	Glycosyl transferase, family 25		
GGTG_11842	10.71	7.84	−2.82	Putative cytochrome P450 monooxygenase		
GGTG_03493	1.95		−2.85	Integral membrane protein		
GGTG_11826	9.68	5.97	−3.66	Cyclohexanone –monooxygenase		
GGTG_11845	7.77		−4.11	Putative ABA3 protein		
GGTG_03814	5.90		−2.92	AhpD-like protein		
GGTG_11844	5.97		−3.62	Putative cytochrome p450 monooxygenase protein/pisatin demethylase protein		

**Ggt*, the comparison of total *Ggt* transcriptome on wheat roots compared to *Ggt* on PDA plate; *Bv+Ggt*, the comparison of total *Ggt* transcriptome on wheat roots pretreated by *Bv* compared to *Ggt* on PDA plates; *Bv+Ggt*_vs_*Ggt*, the comparison of total *Ggt* transcriptome on wheat roots pretreated by *Bv* compared to *Ggt* on wheat roots. “−” represents negative values (downregulation).*

### HCA of DEGs

**FIGURE 1:**
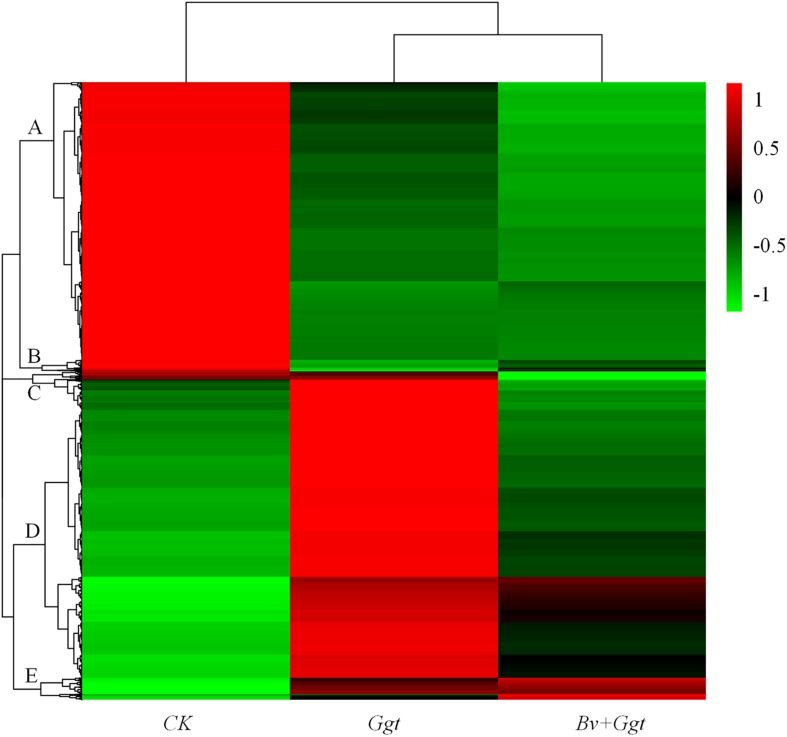
Hierarchical clustering analyses of all DEGs. The analysis was based on three treatments’ RPKM value from the transcriptomic data of *CK*, *Ggt*, and *Bv+Ggt* treatments; red indicates high expression of genes, while green indicates low expression of genes. *CK*, the *Ggt* sample on the PDA plates; *Ggt*, the wheat roots infected with pathogen *Ggt*; *Bv+Ggt*, the *Ggt*-infected wheat roots in the presence of *Bv*.

The HCA was conducted using those 4,260 DEGs that underwent significant changes in their expression in at least one replicate sample. This analysis revealed clear clustering of *Ggt*-infected wheat roots whether precolonized with *Bv* (*Bv+Ggt*) or not (*Ggt*), with the *Ggt* sample on PDA clustered into another branch entirely (Figure 1). Based on the expression levels of the DEGs, gene expression patterns were divided into five groups for the three experimental conditions. Clusters A and D contained the bulk of the DEGs. Cluster A and B were enriched in transcripts showing lower expression levels in *Ggt*-infected wheat roots, regardless of precolonization with *Bv*, versus the *Ggt* on PDA. Cluster C and D contained transcripts that were highly upregulated in the *Ggt*-infected wheat roots but whose upregulation was significantly suppressed in the presence of *Bv* or in *Ggt* on PDA. Cluster E contained those DEGs with very lower expression levels for *Ggt* on PDA while representing a higher expression level in the infected wheat roots with or without *Bv.*

### Functional Enrichment of DEGs

**FIGURE 2:**
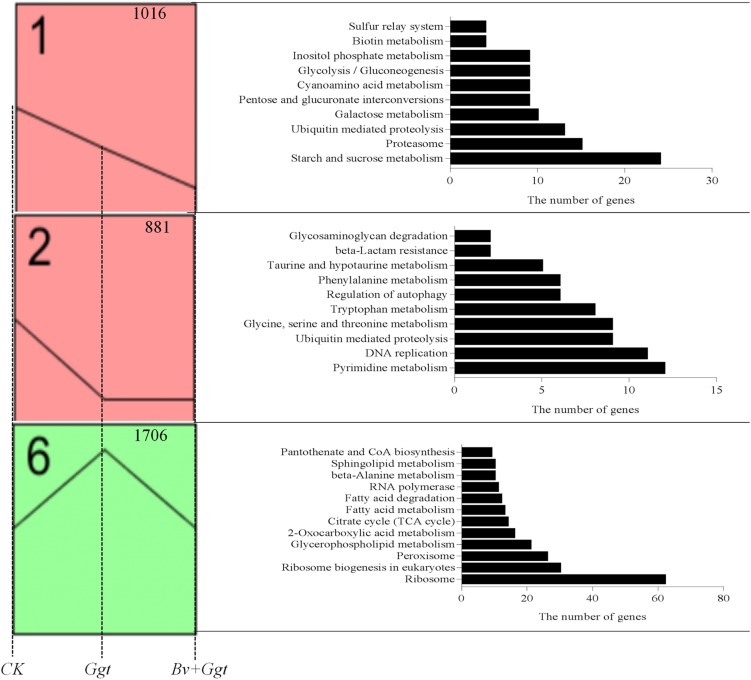
The significant (*P* values < 0.05) expression profiles generated by the analysis of the RPKM value based on the *Ggt* transcriptomic data of *CK*, *Ggt*, and *Bv+Ggt* treatments. The figure at the upper right corner represents the gene number. All significant annotated KEGG pathways for each profile are listed on the right shown by histograms. *CK*, the *Ggt* sample on the PDA plates; *Ggt*, the wheat roots infected with pathogen *Ggt*; *Bv+Ggt*, the *Ggt*-infected wheat roots in the presence of *Bv*.

A total of 4,260 transcripts that showed significant differential expression in at least one sample were used for the STEM analysis, resulting in three significantly enriched expression profiles (Figure 2) out of the eight enriched expression profiles (*P* values <0.05). Profile 1 contained 1,016 transcripts, which were significantly enriched in 10 pathways based on the KEGG database. These pathways were mainly involved in carbohydrate metabolism, such as for starch and sucrose, pentose and glucuronate interconversions, galactose, glycolysis/gluconeogenesis, and inositol phosphate. Compared with *Ggt* on PDA, all of the included DEGs were downregulated in *Ggt* in wheat roots with or without *Bv*. Yet, the expression levels of genes in *Ggt* in the *Bv*+*Ggt*-infected wheat roots were lower than those observed in *Ggt*-infected wheat roots. Profile 2 had 881 genes, enriched in 10 pathways, of which three were related to amino acid metabolism (e.g., taurine and hypotaurine; tryptophan, glycine, serine; and threonine and phenylalanine), while two pathways were involved in DNA replication and pyrimidine metabolism. These genes were downregulated in both the *Ggt-* and the *Bv*+*Ggt*-infected wheat roots relative to *Ggt* on PDA. Profile 6 contained the highest numbers of transcripts (1,706), which were enriched into 12 pathways, including lipid metabolism (e.g., sphingolipid, fatty acid, glycerophospholipid, and fatty acid degradation), tricarboxylic acid cycle, and peroxisome metabolism assigned to primary metabolism. Expression of these genes in *Ggt*-infected wheat roots was much higher than that on PDA plates or in *Bv*+*Ggt*-infected roots; hence, *Bv* might inhibit *Ggt* infection by regulating its primary metabolism.

### DEGs for Secreted Proteins

During the process of host infection, fungi generally secrete a suite of proteins and enzymes to evade or counteract plant defense systems and alter the microenvironment of their host. *Ggt* reportedly harbors 1,001 secreted proteins with structures consisting of signal peptides and cleavage sites, subcellular targeting, transmembrane (TM) spanning regions, and glycosylphosphatidylinositol (GPI) anchor proteins (Xu et al., 2016). When compared to the gene ID of secreted proteins recently predicted by Xu et al. (2016), we identified a total of 458 (372 upregulated, 86 downregulated) and 198 (161 upregulated, 37 downregulated) secreted protein-coding DEGs (Supplementary Figure S3) among the 4,260 DEGs (Supplementary Figure S2A) in *Ggt*- and *Bv*+*Ggt*-infected roots, respectively. This clearly suggested that precolonization by *Bv* significantly regulated the expression of genes associated with secreted proteins in *Ggt* in wheat roots.

**FIGURE 3:**
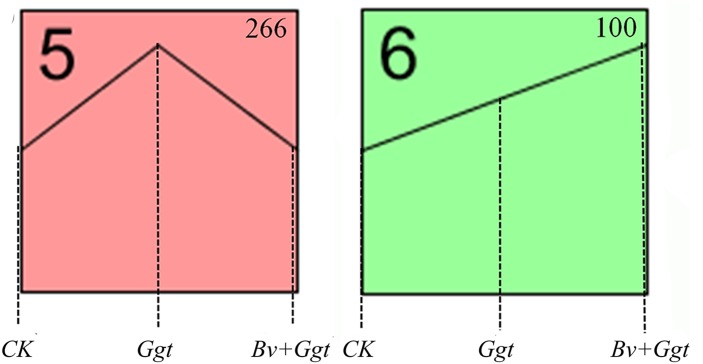
The significant (*P* values < 0.05) expression profiles for identified secreted protein-coding genes. The analysis was based on the RPKM value of the *Ggt* transcriptomic data from *CK*, *Ggt*, and *Bv+Ggt* treatments. *CK*, the *Ggt* sample on the PDA plates; *Ggt*, the wheat roots infected with pathogen *Ggt*; *Bv+Ggt*, the *Ggt*-infected wheat roots in the presence of *Bv*.

The STEM analysis revealed that all 469 DEGs encoding secreted proteins clustered significantly into profiles 5 and 6 (*P* values < 0.05) (Figure 3) but not so (*P* values*>* 0.05) in the other six profiles (not shown). Profile 5 included 266 genes whose expression was activated in *Ggt*-infected wheat roots but were mostly unaltered in the *Bv*+*Ggt*-infected roots or in *Ggt* on PDA (Figure 3). Profile 6 included 100 genes upregulated in the *Ggt-* and *Bv*+*Ggt-*infected wheat roots compared with *Ggt* grown on PDA (Figure 3). Moreover, the genes of both profiles were largely enriched in categories of catalytic activity, carbohydrate binding, and pattern binding. In profiles 5 and 6, a total of 137 genes (37.43%) participated in catalytic activities primarily related to hydrolases and oxidoreductases (Supplementary Tables S3, S4). Among these hypothesized hydrolase genes, most of them encode pectase, cellulase, xylanase, keratase, and peptidase, all of which play key roles in cell wall degradation. All the genes encoding oxidoreductase are capable of peroxidase activity with the function of pathogen self-defense against plant immune response. Additionally, the expression of *Ggt* genes encoding papain inhibitors involved in suppressing host protease activity was distinctly suppressed in *Bv*+*Ggt*-infected wheat roots. Collectively, these results suggested the presence of *Bv* directly and/or indirectly impaired the pathogenicity (e.g., cell wall degradation enzymes, oxidoreductases, and papain inhibitors, among others) of *Ggt* in wheat roots through complex regulatory mechanisms.

### DEGs for Fungal Pathogenicity

**FIGURE 4:**
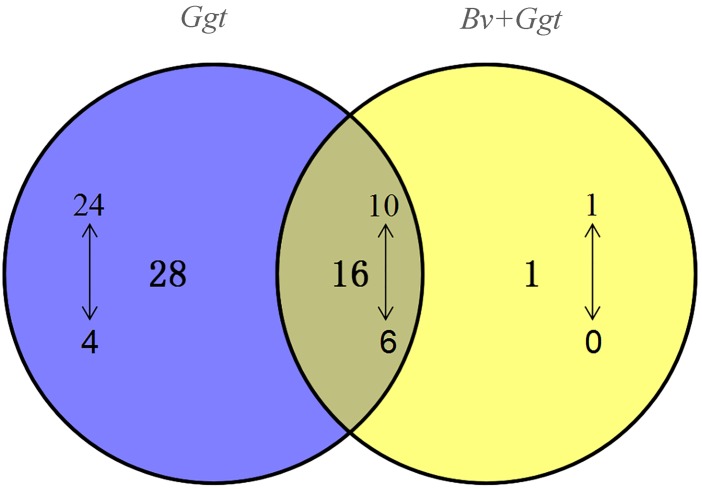
The numbers of DEGs encoding *Ggt* pathogenicity proteins. The Venn diagram illustrates the shared and specific pathogenicity genes generated by the comparative *Ggt* transcriptomic data analysis of *Ggt*-infected wheat roots with or without *Bv* compared to *Ggt* on PDA plates. The upward (or downward)-pointing arrow represents up (or down)-regulated gene expression. *Ggt*, the comparison of total *Ggt* transcriptome on wheat roots compared to *Ggt* on PDA plate; *Bv*+*Ggt*, the comparison of total *Ggt* transcriptome on wheat roots pretreated by *Bv* compared to *Ggt* on PDA plates.

Based on the BLAST analyses of the PHI database, a total of 151 pathogenicity-related genes were identified in the *Ggt* genome (Supplementary Table S5). Of those, 83 genes are recognized as established determinants of pathogenicity in various pathogenic fungi (Supplementary Table S5), for which 44 (34 upregulated, 10 downregulated) were significantly expressed in *Ggt*-infected wheat roots in the absence of *Bv*, whereas 17 (11 upregulated, 6 downregulated) were significantly expressed in *Bv*+*Ggt*-infected wheat roots. The distribution of up- and downregulated *Ggt* pathogenicity DEGs is depicted in the Venn diagram (Figure 4). Evidently, 28 pathogenicity genes were uniquely regulated in *Ggt*-infected wheat roots, of which 4 genes – CTB5, Sc Srb10, ACP, and DEP4 – were downregulated during *Ggt* infection (Supplementary Table S6). Only one gene coding for appressorial penetration-associated GAS2 was found upregulated (2.69 fold change) in *Bv*+*Ggt*-infected wheat roots but not in *Ggt*-infected wheat roots. These results suggested that the presence of the biocontrol bacterium *Bv* reduces the pathogenesis of *Ggt* during its infection of wheat roots.

### qRT-PCR Validation

The expression of the target genes, normalized by three internal reference genes, was downregulated between *Ggt-* and *Bv+Ggt*-infected wheat roots. Comparing the ratio of qRT-PCR expression and RPKM values for the *Bv*+*Ggt*-infected to the *Ggt*-infected wheat roots revealed they were mostly consistent, indicating the RNA-Seq data were robust (Supplementary Table S7).

## Discussion

This study compared the transcriptomes of *Ggt* and *Ggt*-infected wheat root in the absence and presence of the biocontrol bacterium *Bv* using the RNA-Seq platform. A total of 4,134 and 2,011 twofold DEGs were, respectively identified from the *Ggt* in the *Ggt*-infected roots without and with *Bv* relative to *Ggt* grown on PDA plates. Numbers of the upregulated and downregulated DEGs in the *Ggt*-infected roots were, respectively reduced by 55.3% and 47.1% when *Bv* had precolonized wheat roots. Some of these DEGs, whether related to *Ggt* pathogenicity or not, are consistent with those reported by Yang et al. (2015), but many of them were newly identified and, thus, warrant future investigation. However, only 31 DEGs (2 upregulated, 29 downregulated) were detected by directly comparing the *Ggt*- and *Bv*+*Ggt*-infected wheat root libraries (Table 1). These limited numbers of DEGs may truly reflect the direct or indirect (e.g., induced plant resistance) regulation of *Ggt*’s gene expression by endophytic *Bv* in wheat roots. Previous studies have shown that *Bv* can mutually coexist with plants and produce antifungal metabolites such as iturins *in vitro* and *in vivo*, which might have contributed to the changed *Ggt* transcriptome in the infected wheat roots (Gong et al., 2015; Cai et al., 2017; Kang et al., 2018). Moreover, beneficial bacteria impose small impacts upon plant root transcriptomes (Pieterse et al., 2014), and they may impose similarly small impacts upon fungal transcriptomes. This may explain the small number of DEGs observed between *Ggt*- and *Bv*+*Ggt*-infected wheat roots in this study.

Many secretory proteins have crucial functions in the fungal infection process. For example, CWDEs are the major pathogenicity factors involved in plant cell wall breakdown and are secreted by many pathogens during infection, such as *Fusarium graminearum* (Zhang et al., 2012), *Colletotrichum orbiculare* (Gan et al., 2013), *Colletotrichum gloeosporioides* (Alkan et al., 2015), *Zymoseptoria tritici* (McDonald et al., 2015), *Colletotrichum graminicola* (Torres et al., 2016), *Dothistroma septosporum* (Bradshaw et al., 2016), and *Leptosphaeria maculans* (Gervais et al., 2017). For example, the expression of genes encoding CWDEs (e.g., endo-1,4-b-xylanase, glycoside hydrolase family 61) were highly upregulated in *Magnaporthe oryzae*, a rice fungus closely related to the pathogen *Ggt* (Kawahara et al., 2012). Thus, inhibition of CWDE gene expression is one of the mechanisms by which bacteria exert biocontrol *in vitro* (Mela et al., 2011; Gkarmiri et al., 2015). Our *in vivo* test also demonstrated the biocontrol efficacy of *Bv* against *Ggt* might contribute to the inhibition of gene expression encoding CWDEs, since these genes were significantly upregulated in *Ggt*-infected roots but downregulated in *Bv+Ggt*-infected wheat roots compared to *Ggt* grown on PDA plates (Figure 3, Table 1, and Supplementary Tables S3, S4). For instance, the expression of cutinase 1 and xylanase in *Bv*+*Ggt*-infected roots was at least twofold lower than that in *Ggt*-infected roots.

Papain-like cysteine proteases (PLCPs) are a large class of proteolytic enzymes found in most plant species (Misas-Villamil et al., 2016). According to recent studies, a plant can protect itself from pest or pathogen attacks by producing PLCPs (Misas-Villamil et al., 2016; Liu et al., 2018); however, in the long-term battle waged between pathogens and plants, pathogens will evolve a PLCP inhibitor (e.g., EPIC2B and avirulence protein 2) to counteract host-derived PLCPs (Kruger et al., 2002; Tian et al., 2004, 2005, 2007). In line with this, we found that the expression of PLCP inhibitor-encoding genes was upregulated in *Ggt*-infected wheat roots compared with *Ggt* grown on PDA (Figure 3 and Supplementary Table S4), indicating that one or more PLCP inhibitors may play an important role in the process of *Ggt* infection. In stark contrast, the expression of PLCP inhibitor-encoding genes in *Ggt*-infected wheat roots precolonized by *Bv* was reduced substantially (Figure 3 and Supplementary Table S4). This result strongly suggests that low-level expression of PLCP inhibitor-encoding genes may be one of the biocontrol mechanisms exerted by *Bv* toward the fungal pathogen *Ggt.*

As a member of the peroxidases, catalase could mediate the decomposition of hydrogen peroxide into water and molecular oxygen, so as to protect the pathogens from reactive oxygen species (ROS) produced by both fungi themselves and host plants (Gardiner et al., 2015; Mir et al., 2015). Work by Singh et al. (2012) revealed that in the response of the fungal pathogen *Verticillium longisporum* to *Brassica napus* xylem sap, catalase peroxidase (VlCPEA) was the most upregulated protein, whereas knockdowns of VlCPEA-encoding genes resulted in sensitivity against ROS. In our study, many genes with predicted peroxidase activity (e.g., catalase) were found upregulated (Figure 3, Table 1, and Supplementary Table S4) during the pathogen *Ggt*’s infection of wheat roots, a result that agrees with previous reports (Govrin and Levine, 2000; Singh et al., 2012; Bao et al., 2014). This result suggests that, similar to the mechanism underlying other pathogenic infections, catalase peroxidase might also participate in protecting the fungus *Ggt* from the oxidative stress generated by wheat plants (Singh et al., 2012). Yet, because the expression of genes encoding peroxidase (e.g., catalase) was decreased in *Bv*+*Ggt*-infected wheat roots (Figure 3, Table 1, and Supplementary Table S4), the presence of biocontrol bacteria *in planta* could have diminished such detoxification activity by reducing peroxidase secretion.

ABA is a crucial molecule for regulating the growth and development of plants, and their stress responses and pathogenicity, and it has been widely studied (Spence et al., 2015). By examining the role of ABA produced by *M. oryzae* in rice leaves, Spence et al. (2015) suggested that it enhances disease severity in two ways, by increasing plant susceptibility and accelerating the pathogenicity of the pathogen itself. For instance, *Pseudomonas syringae* indirectly utilizes ABA as an effector molecule to modulate endogenous host biosynthesis of ABA, thus perturbing the ABA-mediated host defense responses (Lievens et al., 2017). In this current study, we identified a homologous gene (ABA3) encoding an enzyme involved in ABA biosynthesis (Siewers et al., 2006; Fan et al., 2009). The expression of this gene was 7.77-fold higher in *Ggt*-infected wheat roots but was lowered by almost 50% to being 4.11-fold higher in *Bv*+*Ggt*-infected wheat roots relative to PDA (Table 1). This result suggests that ABA, at least, is likely a critical component in plant–pathogen interactions between wheat and *Ggt*, whereas *Bv* might also use this ABA functioning to impair pathogen infection by disturbing *Ggt*-derived ABA synthesis and thus limiting fungal pathogenesis. Targeting these candidate pathogenicity genes/factor through further experimental analysis, such as gene knockouts in *Ggt*, will enable a better understanding of the biocontrol mechanisms of *Bv* that act on the pathogen *Ggt*.

Many plant pathogenic fungi have evolved the capacity to breach intact cuticles of their plant hosts by using an infection structure called the appressorium (Ryder and Talbot, 2015). Previous studies reported that during infection with a pathogen, the accumulation of glycerol in appressoria/hyphopodia results in highly localized turgor pressure upon the cell wall, and this further assists fungal pathogens to overcome cellular barriers for successful hyphal infection and extension (DeJong et al., 1997). For example, Martin-Urdiroz et al. (2016) have shown that glycerol accumulation of the appressorium in rice blast fungus *Magnaporthe oryzae* drives turgor-mediated penetration of the rice leaf. However, work by Sha et al. (2016) indicated that, *in vitro*, the biocontrol strain *Bacillus subtilis* suppressed the appressorial formation of *Magnaporthe oryzae*. The metabolism of glycerophospholipids, carbohydrate, and peroxisome may have direct or indirect effects on the biosynthesis of glycerol or its precursors’ replenishment (Supplementary Figure S4). As shown in profile 6, the activation or enhancement of the metabolism of glycerol (Figure 2) could be beneficial for *Ggt* to infect wheat roots. Conversely, the metabolism of glycerol was suppressed in *Bv+Ggt-*infected wheat roots. Thus, limiting the expression of glycerol synthesis-related genes in *Ggt* may be among the biocontrol strategies that *Bv* employs *in planta*.

Inexplicably, when compared with *Ggt* on PDA, 10 pathogenicity-related genes were downregulated in *Ggt*-infected wheat roots, and likewise six genes in *Bv+Ggt*-infected wheat root (Figure 4). These genes mainly encode MoAAT (4-aminobuty-rate aminotransferase), Chi2 (endochitinase), avenacinase, Ss-ggt1 (gamma-glutamyl transpeptidase), and SS-odc2 (oxalate decarboxylase). Although we do not know why these pathogenicity-related genes are downregulated in infected wheat roots, plausible reasons include the following: (1) these genes are host dependent and their activation is not essential in the *Ggt*–wheat ecological system; (2) the internal environment of wheat roots is not suitable for the expression of these genes; and (3) these genes are highly expressed in PDA, resulting in relatively low expression in wheat. As to which explanation is most probable and operative, this ought to be tested in the future.

## Conclusion

This study has provided novel insights into a potential pathogenicity mechanism of *Ggt* in wheat roots, whether strain CC09 is present or absent (relative to the *Ggt* grown on PDA plates) through comparative analysis of their transcriptomes using the Illumina platform. Many novel candidate genes related to *Ggt* pathogenesis were identified, and some potential targets of biocontrol bacteria were discussed. The gene expression data presented in this study suggest that the following mechanisms likely play a role in the biocontrol efficacy of *Bv* against *Ggt* in wheat: (a) decreased amount of fungus-derived CWDEs; (b) repressed genes encoding papain inhibitors, catalase-3, and ABA3; and (c) limited hyphopodia formation that impedes pathogen infection. In addition, our results enhance our knowledge of not only the pathogenicity of *Ggt* at the early infection stage in wheat roots but also the potential mechanism of an endophytic biocontrol bacterium *in planta*. Nonetheless, to what extent *Bv*-induced plant defense fosters the biological control effect of *Bv* upon *Ggt* infection remains to be elucidated.

## Data Availability

The datasets generated for this study are available in the National Center for Biotechnology Information, PRJNA 485739 and PRJNA496308.

## Author Contributions

CL, YX, and XK designed the research study. XK, YG, and SL performed the experiments. XK and CL wrote the manuscript and analyzed the data. CL, XK, SL, YG, LX, and LW assisted in structuring and editing the work. All authors contributed substantially to revisions and approved the final manuscript.

## Conflict of Interest Statement

The authors declare that the research was conducted in the absence of any commercial or financial relationships that could be construed as a potential conflict of interest.

## Footnotes

1 http://fungi.ensembl.org/info/website/ftp/index.html

2 http://systemsbiology.cau.edu.cn/agriGOv2/

3 https://www.kegg.jp/

4 http://bioinfogp.cnb.csic.es/tools/venny/index.html

5 http://www.cs.cmu.edu/~jernst/stem/

## Supplementary Material

FIGURE S1 Hierarchical clustering of transcripts for each replicate from *Ggt* and *Bv*+*Ggt* treatments. Hierarchical clustering analysis of the transcriptional profiles was performed using the hclust command in R and the default complete linkage method. Each gene’s expression was Z-score normalized separately within each of the three data sets. Rows (genes) were clustered hierarchically. Columns (RNA samples) were sorted by sample metadata. The genes with higher (red) or lower (blue) expression are represented. *CK*, the *Ggt* sample on the PDA plates; *Ggt*, the wheat roots infected with pathogen *Ggt*; *Bv+Ggt*, the *Ggt*-infected wheat roots in the presence of *Bv*.

FIGURE S2 The DEG expression profile obtained from the comparison of total *Ggt* transcriptome on wheat roots pretreated by *Bv* or not compared to *Ggt* on the PDA plate. **(A)** The number of *Ggt* DEGs in response to *Ggt*- and *Bv*+*Ggt*-infected wheat roots, respectively. **(B)** Venn diagram illustrating the number of *Ggt* DEGs upregulated or downregulated in *Ggt-* and *Bv+Ggt*-infected wheat roots. **(C)** The level of *Ggt* gene expression in response to *Ggt*- and *Bv*+*Ggt*-infected wheat roots, respectively. The expression level is shown along the horizontal axis, while values on the vertical axis indicate the gene number. *CK*, the *Ggt* sample on the PDA plates; *Ggt*, the wheat roots infected with pathogen *Ggt*; *Bv+Ggt*: the *Ggt*-infected wheat roots in the presence of *Bv*.

FIGURE S3 The number of DEGs encoding secreted proteins obtained from the comparison of total *Ggt* transcriptome on wheat roots pretreated by *Bv* or not compared to *Ggt* on the PDA plate.

FIGURE S4 Overview of the glycerol biosynthesis in fungus. The solid lines, dashed lines, circle marks, and frames represent the direct link, indirect links/unknown reaction, chemical compound, and metabolism/enzymes, respectively.

TABLE S1 The selected DEGs and the sequences of primer pairs used for the qRT-PCR experiment.

TABLE S2 Summary of the sequence analysis after Illumina sequencing.

TABLE S3 List of molecular functions for the identified secreted protein-coding genes, based on the data of profile 6 in Figure 3.

TABLE S4 List of molecular functions for identified secreted protein-coding genes, based on the data of profile 5 in Figure 3.

TABLE S5 The pathogenicity-related genes identified in the *Ggt* genome.

TABLE S6 The expression profile of DEGs assigned to pathogenicity proteins.

TABLE S7 Relative expression levels of selected pathogenicity-related DEGs in *Ggt-* and *Bv+Ggt*-infected wheat roots.
