# Supplementary figures and images for: Comparative Transcriptome Profiling of Gaeumannomyces graminis var. tritici in Wheat Roots in the Absence and Presence of Biocontrol Bacillus velezensis CC09
Source: Front Microbiol. 2019 Jul 9;10:1474. doi: 10.3389/fmicb.2019.01474 (PMC6629770; doi:10.3389/fmicb.2019.01474)

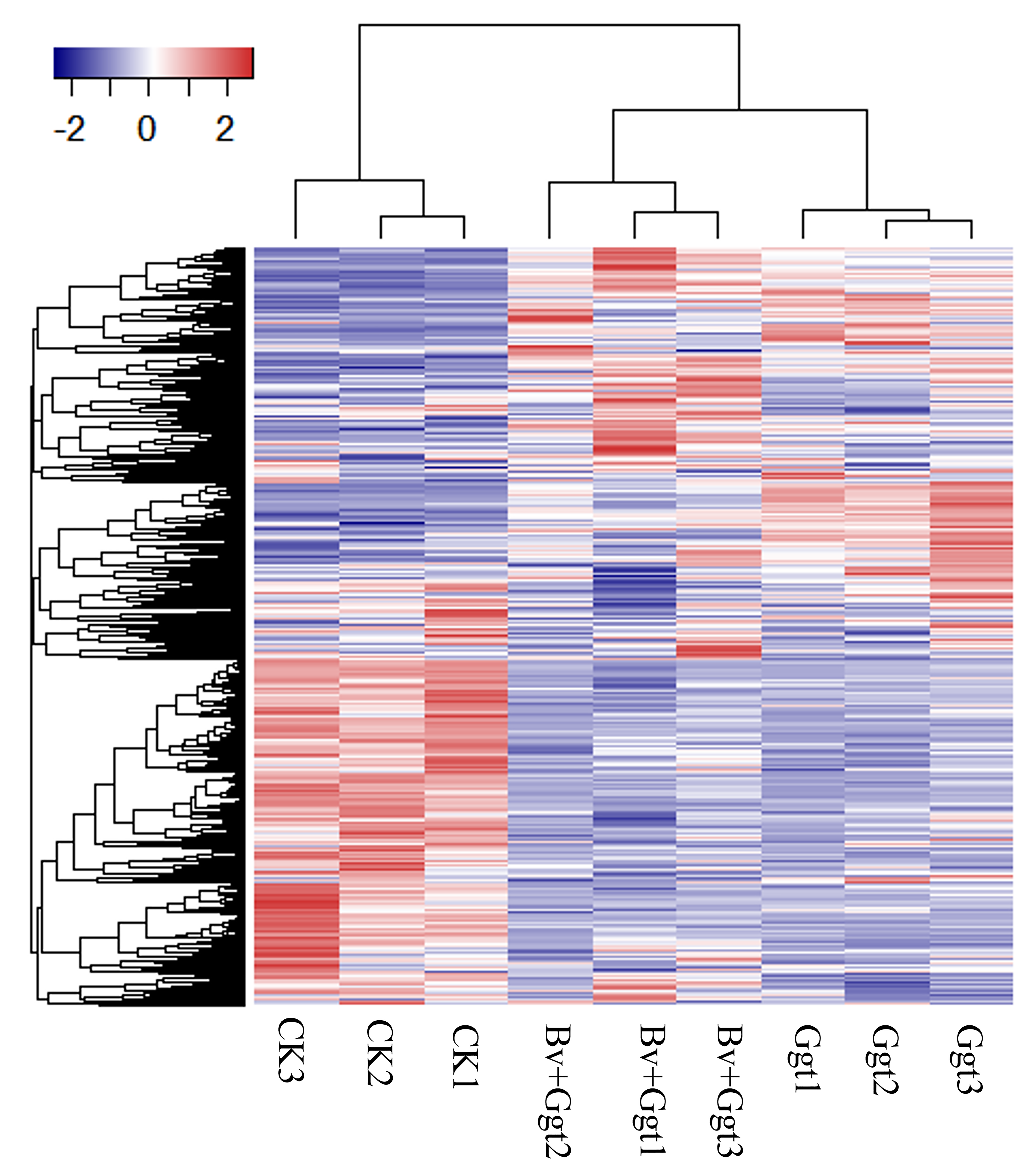

Supplement: FIGURE S1 — Hierarchical clustering of transcripts for each replicate from Ggt and Bv+Ggt treatments. Hierarchical clustering analysis of the transcriptional profiles was performed using the hclust command in R and the default complete linkage method. Each gene’s expression was Z-score normalized separately within each of the three data sets. Rows (genes) were clustered hierarchically. Columns (RNA samples) were sorted by sample metadata. The genes with higher (red) or lower (blue) expression are represented. CK, the Ggt sample on the PDA plates; Ggt, the wheat roots infected with pathogen Ggt; Bv+Ggt, the Ggt-infected wheat roots in the presence of Bv. [file Image_1.TIF]

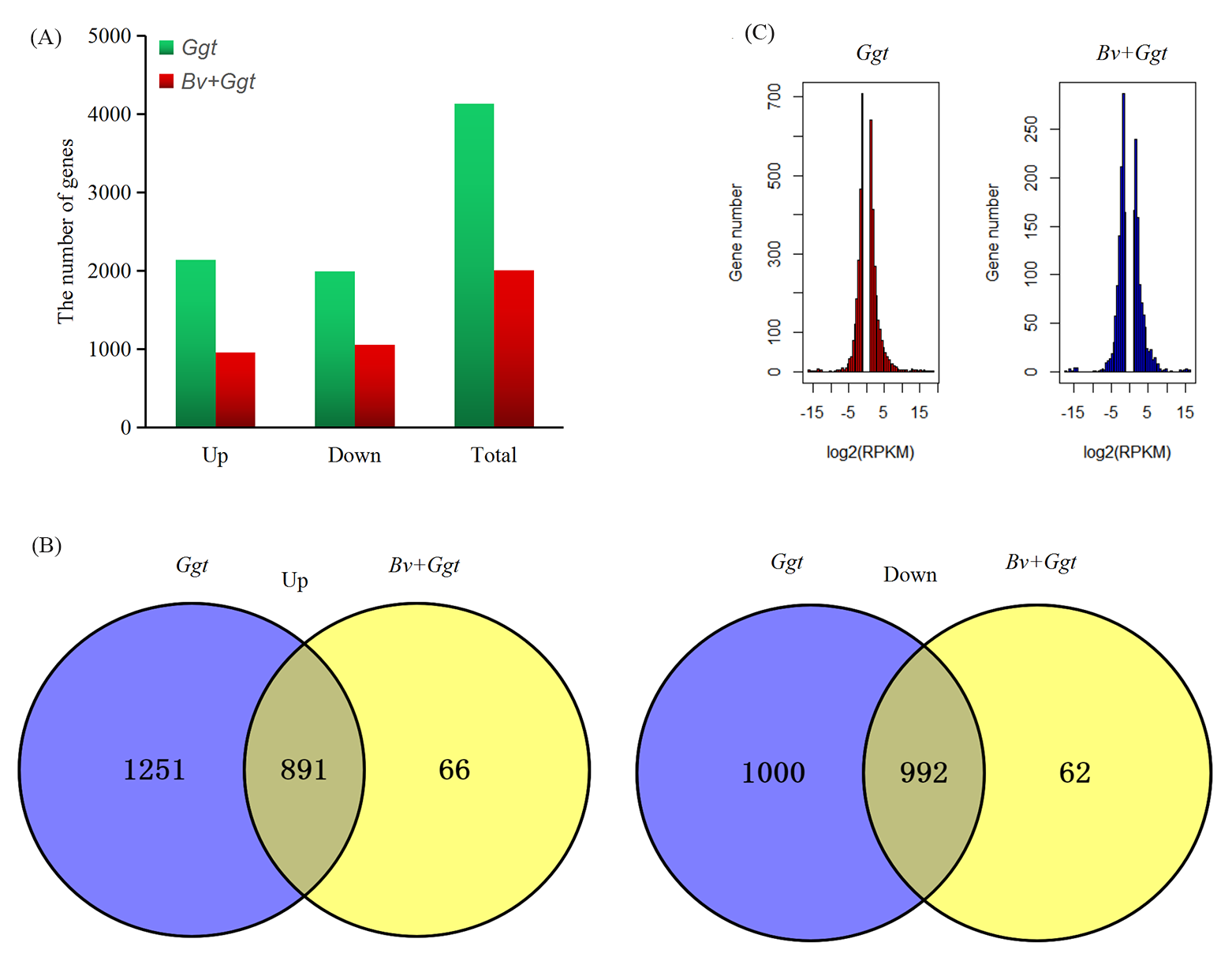

Supplement: FIGURE S2 — The DEG expression profile obtained from the comparison of total Ggt transcriptome on wheat roots pretreated by Bv or not compared to Ggt on the PDA plate. (A) The number of Ggt DEGs in response to Ggt- and Bv+Ggt-infected wheat roots, respectively. (B) Venn diagram illustrating the number of Ggt DEGs upregulated or downregulated in Ggt- and Bv+Ggt-infected wheat roots. (C) The level of Ggt gene expression in response to Ggt- and Bv+Ggt-infected wheat roots, respectively. The expression level is shown along the horizontal axis, while values on the vertical axis indicate the gene number. CK, the Ggt sample on the PDA plates; Ggt, the wheat roots infected with pathogen Ggt; Bv+Ggt: the Ggt-infected wheat roots in the presence of Bv. [file Image_2.TIF]

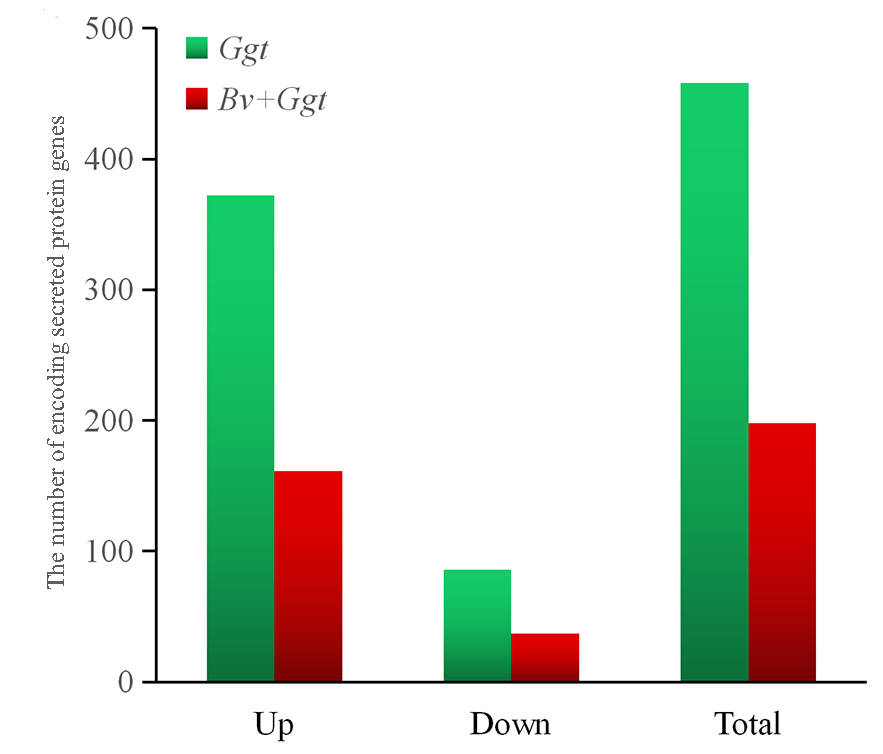

Supplement: FIGURE S3 — The number of DEGs encoding secreted proteins obtained from the comparison of total Ggt transcriptome on wheat roots pretreated by Bv or not compared to Ggt on the PDA plate. [file Image_3.TIF]

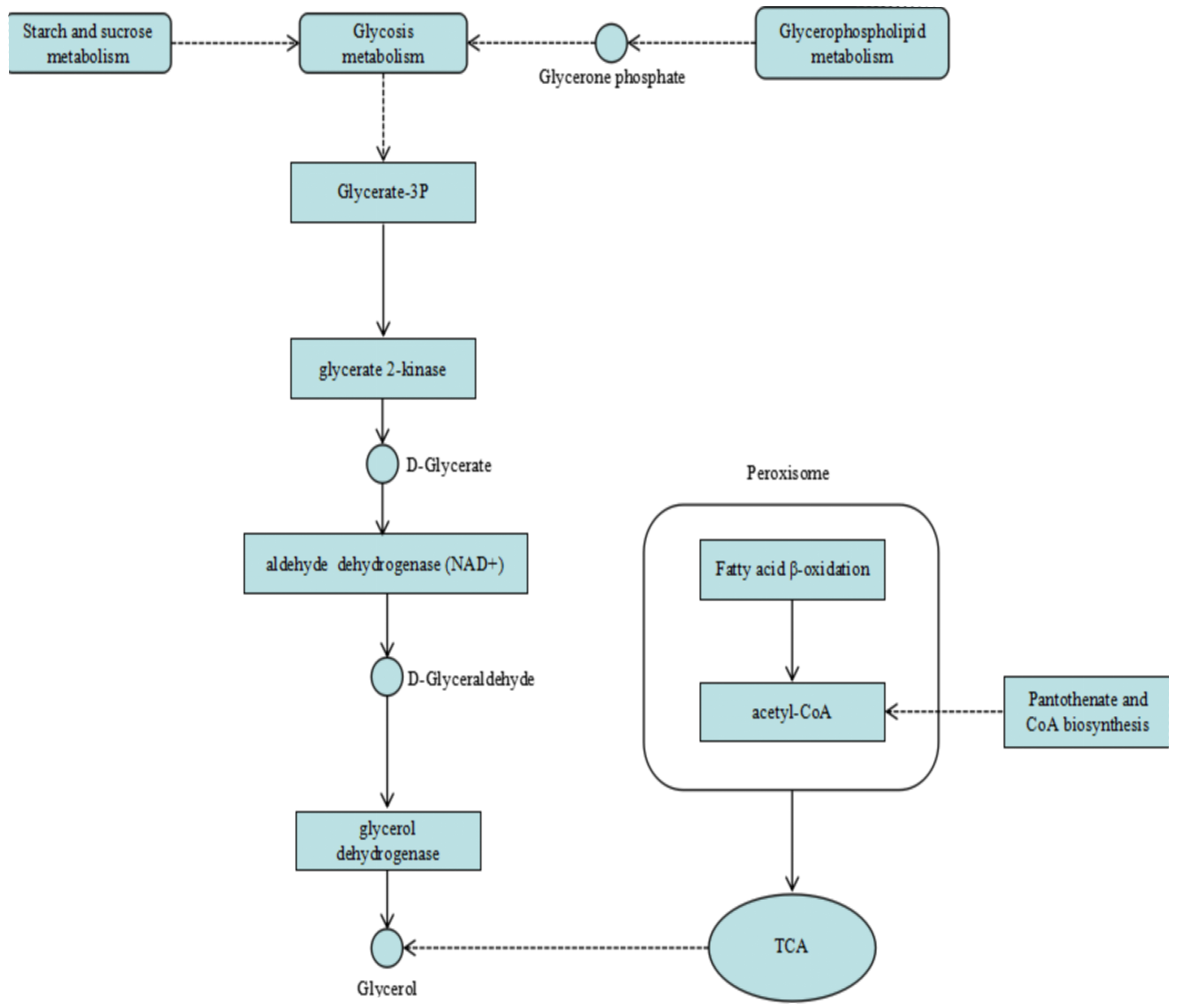

Supplement: FIGURE S4 — Overview of the glycerol biosynthesis in fungus. The solid lines, dashed lines, circle marks, and frames represent the direct link, indirect links/unknown reaction, chemical compound, and metabolism/enzymes, respectively. [file Image_4.TIF]
